# Enhanced Neural Responses to Self-Name Stimuli Relative to Tone and Reversed Speech Deviants in the Auditory Oddball Paradigm

**DOI:** 10.3390/brainsci16060608

**Published:** 2026-06-02

**Authors:** Fang Duan, Xiongping Cao, Zheng Yan, Jianming Chen

**Affiliations:** College of Information Science and Engineering, Huaqiao University, Xiamen 361021, China; 23013082042@stu.hqu.edu.cn (X.C.); zhengyan_bme@hqu.edu.cn (Z.Y.); 23014082003@stu.hqu.edu.cn (J.C.)

**Keywords:** auditory oddball paradigm, event-related potential, source reconstruction, self-referential processing, EEGNet, neural discriminability

## Abstract

**Highlights:**

**What are the main findings?**

- Self-referential auditory stimuli (one’s own name) elicit significantly more robust neural signatures, including higher P300 amplitudes (3.95 μV), than simple tones (1.77 μV).
- Source-space analysis revealed that self-name processing recruits a distributed network encompassing salience-processing and self-referential regions, involving 12 significant clusters, whereas acoustic deviance is more localized.

**What are the implications of the main findings?**

- The high neural discriminability of self-name responses (approx. 80% accuracy) suggests its potential utility for auditory paradigm design in BCI and clinical assessment research contexts, pending validation in target populations.
- These results offer a comparative framework for understanding how different dimensions of auditory relevance modulate neural processing, informing the design of effective paradigms for cognitive neuroscience.

**Abstract:**

**Background**: Auditory oddball paradigms are widely used to investigate neural responses to deviant stimuli and attentional processing. However, different paradigms involve deviant stimuli with varying levels of stimulus relevance, and the corresponding neural responses have rarely been directly compared within a unified experimental framework. The aim of this study was to compare neural responses elicited by three variants of the auditory oddball paradigm that differ in the type of deviant stimuli: tone, reversed speech, and self-name deviants. **Methods**: Electroencephalography (EEG) data were recorded from 38 healthy participants while they performed three paradigm variants. Event-related potentials (ERPs) were analyzed to examine neural responses to deviant stimuli. In addition, cortical activation patterns were identified via source reconstruction, and classification analyses were conducted to assess the discriminability of neural responses across the three variants. **Results**: ERP results revealed that the self-name paradigm elicited the largest ERP responses, characterized by a significant P300 amplitude (3.95 μV) and prominent MMN (−6.39 μV). Crucially, source-space analysis revealed a graded expansion of cortical recruitment: acoustic deviance (tone) and structural reanalysis (reversed speech) were associated with 7 and 6 significant clusters, respectively, primarily in the auditory and fronto-cingulate cortices, whereas the self-name paradigm engaged 12 significant clusters spanning a distributed network encompassing salience-processing regions and cortical midline structures associated with self-referential processing (including the insula and posterior cingulate cortex). Classification analyses mirrored these findings, with the self-name paradigm consistently yielding the highest neural separability (~80% accuracy) and greater robustness to interindividual variability, demonstrating the superior discriminability of self-referential neural patterns. **Conclusions**: These findings demonstrate that self-referential auditory stimuli elicit stronger and more discriminable neural responses than other auditory deviant stimuli in the oddball paradigm. These results provide a comparative perspective on how different dimensions of auditory relevance modulate neural processing and may inform the design of effective auditory paradigms for cognitive neuroscience and related translational applications.

## 1. Introduction

Auditory perception is a fundamental component of human cognition, supporting the detection of environmental changes and the allocation of attentional resources to behaviorally relevant stimuli [1,2]. One of the most widely used experimental paradigms for investigating these processes is the auditory oddball paradigm, in which infrequent deviant stimuli are embedded in a sequence of frequent standard stimuli [3]. Deviant stimuli typically elicit characteristic neural responses, including event-related potential (ERP) components such as the mismatch negativity (MMN) and P300, which reflect processes underlying deviance detection, attentional allocation, and stimulus evaluation [4,5].

Variants of the auditory oddball paradigm incorporate deviant stimuli that differ in various stimulus dimensions [6,7]. In the classical tone oddball paradigm, deviant stimuli are typically defined by simple acoustic features (e.g., pitch and frequency) [8,9,10]. In contrast, speech-based oddball paradigms incorporate deviant stimuli in more complex auditory signals, such as alternations in phonetic structure or speech manipulation [11]. Temporally reversed speech has been used to dissociate low-level acoustic processing from lexical–semantic comprehension because it preserves certain acoustic and spectrotemporal properties of speech while disrupting the normal temporal structure required for intelligibility [12,13]. In the present study, reversed speech was therefore included as an intermediate speech-based deviant: it is acoustically more complex than pure tones but lacks the personal salience and clear semantic relevance of one’s own name. Another line of research has focused on self-referential stimuli, which are known to have strong attentional and cognitive relevance [14,15,16,17]. Distinct from prior work, the present study provides a systematic multi-level comparison across three distinct auditory oddball variants—ranging from simple acoustic to complex speech-based and self-referential deviants—within the same cohort. Furthermore, we move beyond traditional scalp-level ERP analysis by integrating 3D cortical source localization and deep-learning-based explainability analysis to provide a more comprehensive spatiotemporal mapping of the neural mechanisms underlying self-referential processing. These distinct types of auditory deviant stimuli engage distinct neural processing pathways via their unique stimulus properties and levels of behavioral relevance [18,19,20].

Previous studies have extensively investigated neural responses in individual variants of the oddball paradigm [21]. For example, the tone oddball paradigm has been widely used to examine fundamental mechanisms of auditory change detection, whereas speech-based paradigms are commonly used to investigate linguistic and semantic processing [22]. Self-name paradigms have garnered increasing attention due to the high salience of self-related stimuli [23,24,25,26,27,28]. However, most existing studies focus on a single paradigm variant, and direct comparisons of neural responses to different types of auditory deviant stimuli in a unified experimental framework remain relatively scarce [29,30,31]. Consequently, it remains unclear how neural responses differ across paradigm variants that vary in terms of stimulus behavioral relevance and complexity.

To address this gap, the present study compared neural responses elicited by three variants of the auditory oddball paradigm that differ in the type of deviant stimuli: tone, reversed speech, and self-name deviants. Electroencephalography (EEG) data were collected from healthy human participants while they completed all three paradigm variants. Event-related potentials (ERPs) were analyzed to characterize neural responses to deviant stimuli. In addition, cortical activation patterns were identified via source reconstruction, and classification analyses were conducted to assess the discriminability of neural responses across the three variants. By comparing these paradigms in a unified experimental design, this study aims to provide a comparative perspective on how different types of auditory deviant stimuli modulate neural responses.

## 2. Materials and Methods

### 2.1. Participants

Thirty-eight healthy right-handed adults (23 males, 15 females; mean age = 23.6 ± 1.9 years) took part in the experiment. All participants completed all three auditory oddball paradigms in a within-subject design. The three paradigms were recorded in separate blocks within the same experimental session. The EEG cap remained on throughout the recording session, with short breaks between blocks; electrode impedances were checked before each block and maintained below 5 kΩ. All participants had normal hearing and normal or corrected-to-normal visual acuity, and no self-reported history of neurological or psychiatric disorders. Written informed consent was obtained from all participants prior to study participation, and the study protocol was approved by the Ethics Committee of the School of Medicine, Huaqiao University. Participants were instructed to abstain from caffeine and alcohol on the day of testing and to obtain sufficient rest to ensure high EEG data quality.

### 2.2. Experimental Design and Stimuli

**Figure 1:**
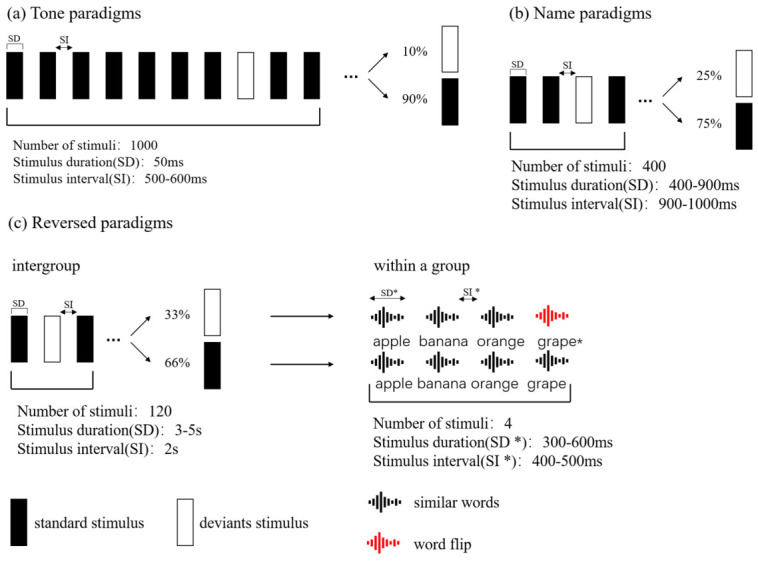
This study employed three experimental paradigms: (**a**) In the Tone paradigm, frequent standard tones (1000 Hz) and infrequent deviant tones (1200 Hz) were presented at a 9:1 ratio under passive listening; (**b**) In the Name paradigm, unfamiliar names served as standard stimuli and the participant’s own name served as the deviant stimulus (3:1 ratio), also under passive listening; (**c**) In the Reversed paradigm, the deviant stimulus consisted of a time-reversed version of the original speech waveform, which preserved the overall acoustic complexity of speech while disrupting its normal temporal structure and semantic intelligibility. Participants silently counted the deviant occurrences (2:1 ratio). In the Reversed paradigm, SD* and SI* indicate the stimulus duration and stimulus interval within a word group, respectively, to distinguish them from the intergroup SD and SI. The asterisk in grape* indicates that the stimulus was the time-reversed audio of “grape”.

Three variants of the auditory oddball paradigm (tone, self-name, and reversed speech) were employed to characterize neural responses across acoustic, semantic, and self-referential dimensions, respectively. To ensure high data quality and mitigate participant fatigue, the parameters of each paradigm variant were optimized via pre-experimental pilot tests to maintain a consistent recording duration of approximately 15 min per paradigm. This standardized duration was prioritized over a strict equalization of stimulus ratios or inter-stimulus intervals (ISI) to address several practical and methodological constraints. First, the inherent physical durations of the stimuli varied significantly (e.g., 50 ms for tones versus 400–900 ms for names); applying a uniform ISI would have resulted in vastly different session lengths, potentially introducing confounding effects related to varying levels of time-on-task fatigue. Second, fixing the total session time allowed for a reasonable balance between collecting a sufficient number of trials for stable ERP averaging and maintaining a stable attentional state. Thus, the 15-min window represents a pragmatic compromise intended to facilitate a cross-paradigm comparison under relatively consistent levels of participant alertness. The experimental paradigm is shown in Figure 1.

The tone paradigm was designed to assess basic sensory deviance detection. Frequent standard tones (1000 Hz) and infrequent deviant tones (1200 Hz) were presented at a 9:1 ratio (1000 trials total) with a 50 ms duration and a 500–600 ms interstimulus interval (ISI) under passive listening conditions.

The self-name paradigm was designed to assess self-referential processing. Unfamiliar proper names were used as standard stimuli, whereas each participant’s own name was used as the deviant stimulus (3:1 ratio; 400 trials total). Stimuli had a duration of 400–900 ms with 900–1000 ms ISI under passive listening conditions.

The reversed speech paradigm was designed to probe the neural mechanisms underlying the structural and semantic reanalysis of speech. Each trial consisted of a four-word meaningful semantic sequence, with the final word of the deviant sequence being time-reversed. This manipulation preserved the overall acoustic complexity of the speech signal while disrupting its normal temporal structure, thereby reducing intelligibility and semantic access. The stimuli had a duration of 300–600 ms, with an ISI of 400–500 ms within each sequence and a 2000 ms inter-sequence interval (120 sequences total; 2:1 ratio).

Notably, unlike the passive listening conditions utilized in the tone and self-name paradigms, participants were instructed to silently count the deviant stimuli in this paradigm. This active task was implemented based on pilot observations suggesting that the reversed speech discrimination task is significantly more cognitively demanding than simple auditory change detection. According to the integrative theory of the P300 [2], the P300 amplitude is highly sensitive to the amount of attentional resources engaged during task performance. Since reversed speech requires complex phonetic analysis without providing immediate semantic reinforcement, it is more prone to attentional drifting under passive conditions. Therefore, the counting task was employed to ensure consistent attentional engagement and to facilitate the reliable elicitation of neural responses by formalizing the resource allocation required for these complex auditory sequences. To verify attentional engagement, participants were asked to verbally report the total count of deviant stimuli at the end of the session. Counting accuracy was quantified as: Accuracy (%) = (1 − |reported count − actual count|/actual count) × 100.

### 2.3. EEG Recording and Preprocessing

**Figure 2:**
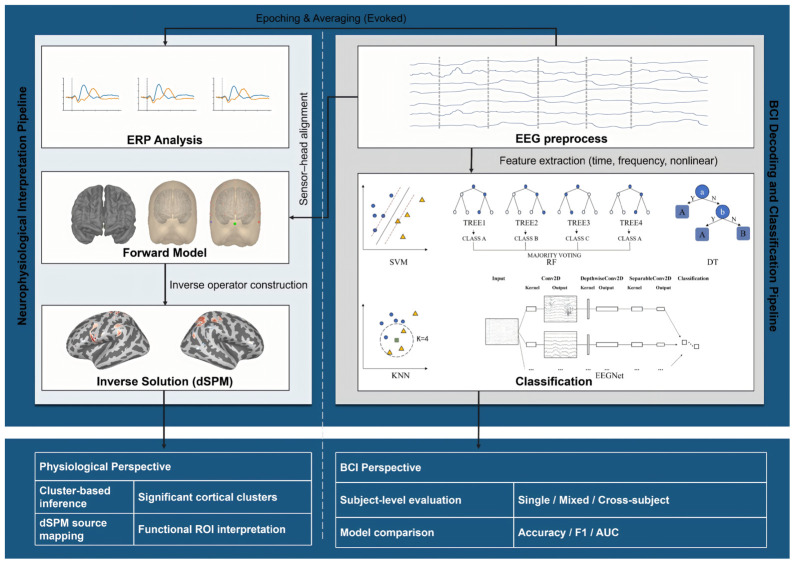
EEG data were preprocessed and epoched to obtain evoked responses. Two complementary analysis pipelines were conducted. Left: Neurophysiological interpretation pipeline. Evoked responses were analyzed at the scalp level (ERP analysis), followed by forward modeling and dSPM inverse reconstruction. Significant cortical activations were identified using cluster-based permutation inference, and activation patterns were interpreted at the ROI level. Right: BCI decoding and classification pipeline. Preprocessed EEG segments were used for time-, frequency-, and nonlinear-domain feature extraction, followed by classification using machine-learning (SVM, RF, KNN, DT) and deep-learning (EEGNet) models. Model performance was evaluated at the single-subject, mixed-subject, and cross-subject levels using accuracy, F1, and AUC metrics. Together, the two pipelines provide complementary neurofunctional interpretation and decoding-based discriminability assessment.

EEG data were recorded using a 68-channel SynAmps^2^ amplifier system (NeuroScan, Charlotte, NC, USA) with Ag/AgCl electrodes placed according to the international 10–20 system. To ensure the analysis focused exclusively on cerebral activity, non-cortical channels—specifically the bilateral mastoids (M1 and M2), the inferior cerebellar electrodes (CB1 and CB2), and the electrooculogram channels (VEO and HEO)—were excluded from all subsequent procedures. Following the removal of these extracerebral artifacts, a total of 60 cortical electrodes were retained for spatial feature extraction and source reconstruction. The final electrode montage consisted of: FP1, FPZ, FP2, AF3, AF4, F7, F5, F3, F1, FZ, F2, F4, F6, F8, FT7, FC5, FC3, FC1, FCZ, FC2, FC4, FC6, FT8, T7, C5, C3, C1, CZ, C2, C4, C6, T8, TP7, CP5, CP3, CP1, CPZ, CP2, CP4, CP6, TP8, P7, P5, P3, P1, PZ, P2, P4, P6, P8, PO7, PO5, PO3, POZ, PO4, PO6, PO8, O1, OZ, and O2. The sampling rate was set to 1000 Hz, and electrode impedance was kept below 5 kΩ for the entire duration of the experiment. Stimulus presentation and trigger synchronization were controlled using PsychoPy version 2023.2.3, which ensured precise event marking for all standard and deviant trials [32]. The technical route of this study is shown in Figure 2.

EEG preprocessing was performed in Python (version 3.8.20) using the MNE-Python toolbox. Continuous EEG signals were first band-pass-filtered from 0.1 to 40 Hz using a zero-phase finite impulse response filter and notch-filtered at 50 Hz to eliminate power-line interference [33]. To establish a neutral reference for subsequent source-space analyses and feature extraction, all scalp EEG data were re-referenced to the common average reference. Independent Component Analysis (ICA) via the FastICA algorithm was subsequently applied to decompose the signals into independent neurophysiological and artifactual components. Artifact identification was conducted by calculating the Pearson correlation coefficients between each independent component and the electrooculography (EOG) channels (horizontal EOG and vertical EOG). Components that exceeded a predefined correlation threshold, reflecting eye blinks or horizontal eye movements, were identified and systematically removed.

Following artifact correction, the preprocessed EEG data were segmented into epochs time-locked to stimulus onset [34,35,36]. Baseline correction was applied using the pre-stimulus interval (−100 to 0 ms relative to stimulus onset). To further ensure high data quality, a semi-automated epoch rejection procedure was implemented: electrodes with amplitudes exceeding ±3 standard deviations (SD) from the grand mean were flagged as abnormal, and any epoch containing more than 10% abnormal electrodes was excluded from subsequent analyses. This rigorous screening procedure effectively eliminated residual noise arising from poor electrode contact or transient movement artifacts, ensuring that only high-quality EEG segments were used for neurophysiological interpretation and classification analyses.

### 2.4. Feature Extraction

To comprehensively characterize neural dynamics across paradigms, EEG features were extracted from three complementary domains: time, frequency, and nonlinear dynamics [37,38,39]. This multidimensional feature extraction approach was designed to capture both canonical ERP features and the complex, non-stationary temporal patterns underlying auditory processing. The inclusion of nonlinear measures was specifically intended to quantify the increased dynamic signal complexity and stability elicited by highly salient stimuli (e.g., one’s own name), which traditional linear analyses may fail to capture. Random oversampling was applied to balance the class distribution of standard and deviant trials in the model training set. It should be noted that random oversampling is only applied to the samples to address the issue of class imbalance, which may slightly overestimate the absolute performance evaluation results. Therefore, the reported absolute classification accuracy should be interpreted with caution.

#### 2.4.1. Time-Domain Features

ERP components were quantified at midline electrodes (Fz, Cz, Pz), which are established recording sites for the MMN and P300 components [2]. Peak amplitudes and latencies were extracted within predefined time windows: MMN within 50–280 ms post-stimulus onset and P300 within 250–450 ms. These windows were selected a priori based on previously reported latency ranges for auditory MMN and P300 components and were broad enough to accommodate paradigm-dependent latency variability across simple acoustic, speech-based, and self-referential stimuli [2,40]. Midline electrodes were selected a priori based on established scalp topographies, thereby avoiding the need for correction across multiple electrodes. Importantly, the remaining cortical electrodes were not excluded from the study; the broader electrode montage was retained for preprocessing, spatial feature extraction, source reconstruction, and classification analyses. Repeated-measures ANOVAs were conducted separately for each ERP metric, as MMN and P300 index distinct neurophysiological processes. Post hoc pairwise comparisons were Bonferroni-corrected for the three paradigm contrasts.

#### 2.4.2. Frequency-Domain Features

Spectral analysis was conducted using a multitaper method to estimate the power spectral density (PSD). The relative power of five canonical EEG frequency bands—delta (1–4 Hz), theta (4–8 Hz), alpha (8–13 Hz), beta (13–30 Hz), and gamma (30–40 Hz)—was calculated for each epoch. Previous EEG studies have shown that oscillatory activity in theta and alpha bands is closely related to attentional engagement, cognitive control, and cortical arousal, with theta enhancement and alpha modulation commonly observed during increased cognitive processing demands [41]. Therefore, to further assess functional neural modulation, band-ratio metrics (i.e., θ/α and (δ + θ)/(α + β)) were calculated as complementary indices associated with attentional allocation, arousal state, and cognitive workload [42,43]. These indices were used to characterize paradigm-specific oscillatory patterns and potential cognitive load differences across paradigms.

#### 2.4.3. Nonlinear Features

Given the nonlinear and nonstationary nature of EEG, five complexity metrics were calculated to capture dynamic signal irregularity: Approximate Entropy (ApEn), Sample Entropy (SampEn), Permutation Entropy (PermEn), Spectral Entropy (SpecEn), and Lempel–Ziv Complexity (LZC). These measures provide complementary indices of EEG signal complexity and irregularity: ApEn and SampEn estimate the regularity of time-series fluctuations, PermEn characterizes the ordinal complexity of temporal patterns, SpecEn reflects the distributional complexity of spectral power, and LZC estimates sequence complexity based on signal compressibility [44,45]. The nonlinear indices were averaged across all scalp sites to derive a representative global measure of temporal complexity for each epoch. It should be emphasized that while this averaging procedure collapses spatial specificity for these particular metrics, the spatial patterns and electrode-level contributions were independently addressed in other parts of our study. Specifically, we performed dSPM-based source reconstruction to map the three-dimensional cortical origins of the auditory responses, and employed Integrated Gradients (IG) to identify significant electrodes and sensor-level topographical contributors within the EEGNet framework. This approach ensures that both global temporal dynamics and localized spatial information are comprehensively investigated.

By integrating time-, frequency-, and nonlinear-domain features, the present study aimed to characterize both the structured ERP components and the intrinsic dynamic characteristics of the brain, yielding a comprehensive multiscale feature set for subsequent classification and neurophysiological interpretation.

### 2.5. Classification Analysis

To assess the discriminability and representational stability of neural responses across paradigms, both traditional machine learning algorithms and a deep-learning framework were employed. The aim was to determine how different dimensions of auditory deviance modulate the separability of standard and deviant trials within a high-dimensional feature space [46].

To assess model robustness and generalizability to interindividual variability, three evaluation schemes were implemented for all classifiers: (1) single-subject evaluation, where models were independently trained and tested for each participant to capture individual-level discriminability; (2) mixed-subject evaluation, which pooled data from all participants to reflect group-level performance; and (3) cross-subject evaluation, which utilized a repeated random subject partitioning (70% training, 30% testing, repeated five times with independent splits) to assess the model’s ability to generalize to unseen neural response patterns.

Model performance was quantified using accuracy, precision, recall, F1-score, and the area under the receiver operating characteristic curve (AUC). Recall (also referred to as sensitivity) measures the proportion of actual deviant stimuli that were correctly identified by the model, serving as a critical indicator of the system’s effectiveness in detecting rare target events. In the context of the auditory oddball paradigm, a high recall ensures that the neural signatures of deviant stimuli are not overlooked among the more frequent standard stimuli. The F1-score is defined as the harmonic mean of precision and recall, providing a balanced assessment of a model’s ability to identify deviant stimuli while minimizing false positives, which is particularly robust in the presence of class imbalance. The AUC represents the probability that a classifier will rank a randomly chosen positive instance higher than a randomly chosen negative one, offering a threshold-independent measure of the model’s overall discriminative power. Given the inherent class imbalance in oddball paradigms, random oversampling was applied to the training set to balance the class distribution of standard and deviant trials, ensuring that these complementary metrics provide a comprehensive evaluation of neural discriminability.

#### 2.5.1. Machine-Learning Models

Four classic machine learning algorithms were employed: Random Forest (RF), Support Vector Machine (SVM), K-Nearest Neighbor (KNN), and Decision Tree (DT). Prior to training, all features were standardized using z-score normalization based on the statistics of the training set. Model hyperparameters were optimized via three-fold cross-validation on the training data to prevent information leakage. The RF consisted of 500 decision trees, the SVM employed a radial basis function kernel, and KNN was set to k = 5 nearest neighbors. Each classifier generated probabilistic outputs corresponding to standard and deviant stimuli.

For cross-subject evaluation, subjects were randomly partitioned into training (n = 27, 70%) and test (n = 11, 30%) sets at the subject level. This partitioning was repeated five times with independent random subject splits to ensure stability of performance estimates, yielding 55 independent test observations per paradigm. Z-score normalization parameters were computed exclusively from the training set and subsequently applied to the test set without re-fitting. Hyperparameter optimization was conducted via Bayesian search within the training set only, and the test set remained entirely held-out throughout all preprocessing and optimization procedures.

#### 2.5.2. Deep-Learning Model

For end-to-end feature learning and classification, the EEGNet architecture was adopted as the deep learning model [47]. The EEGNet architecture was employed to learn discriminative spatiotemporal patterns from preprocessed EEG epochs. Specifically, the model utilizes a temporal convolution to extract frequency features, followed by a depthwise convolution that operates across electrodes to capture spatial patterns. Crucially, these layers do not treat spatial and temporal information as isolated components; instead, the subsequent separable convolutions enable the model to learn the evolving spatial distribution of neural activity over time. This ensures that the dynamic, spatiotemporal dependencies inherent in ERP components (e.g., the shifting topography from MMN to P300) are kept combined and intact throughout the feature extraction process. Input data were band-pass-filtered and downsampled to 128 Hz prior to training. The EEGNet model was optimized using the Adam algorithm with a learning rate of 0.001 and cross-entropy loss. Dropout regularization (*p* = 0.5) and early stopping were applied to mitigate model overfitting, and each training session was repeated five times with random weight initialization to ensure reliability.

For cross-subject evaluation, the same repeated random subject partitioning scheme was applied (27 training, 11 test subjects, 70/30 split, repeated five times with independent subject partitions), yielding 55 independent test observations per paradigm. Unlike the machine learning pipeline, trial-wise z-score standardization was computed independently for each trial, ensuring no statistical dependency between training and test data. Model weights were re-initialized at each repeat to prevent any carry-over of learned representations between runs.

#### 2.5.3. EEGNet-Based Saliency Analysis

To further elucidate the spatial–temporal neural patterns underlying EEGNet classification, an explainability analysis was conducted using the Integrated Gradients (IG) method. IG quantifies the contribution of each input feature by integrating gradients along a path from a baseline input (all-zero signal) to the actual EEG signal, thereby providing a principled attribution of model predictions.

For each paradigm, IG was computed on correctly classified deviant trials using the trained EEGNet model under the cross-subject evaluation scheme. Attribution maps were calculated at the single-trial level and subsequently averaged across trials and subjects to obtain group-level spatiotemporal representations.

A sliding-window approach (50 ms window width) was applied within the 50–450 ms post-stimulus interval to characterize temporal dynamics. Within each window, a non-parametric permutation-based one-sample *t*-test (1000 iterations) was performed across subjects for each channel to test the null hypothesis that an electrode’s contribution was no different from the spatial average. To account for multiple comparisons across the sensor space, *p*-values were adjusted using false discovery rate (FDR) correction (α = 0.05). Significant electrodes were marked on the topographic maps to highlight stable discriminative regions utilized by the model for classification.

This analysis provides an interpretable link between deep-learning-based decoding performance and underlying neurophysiological processes.

### 2.6. Source-Space Estimation

To identify the cortical neural generators of ERP components, source-space analysis was performed using the MNE-Python toolbox. A three-layer boundary element model (BEM) was constructed based on the fsaverage standard brain template. Although the use of a template brain introduces anatomical constraints relative to individual MRI-based models, it provides a robust and standardized framework for group-level inference. Conductivity values were set to 0.3 S/m, 0.006 S/m, and 0.3 S/m for the inner skull, outer skull, and scalp compartments, respectively. Electrode positions were co-registered using fiducial landmarks [48,49].

The inverse solution was calculated using dynamic statistical parametric mapping (dSPM) with a loose orientation constraint (0.2) and a signal-to-noise ratio (SNR) set to 3. The resulting dSPM values represent unitless, noise-normalized statistical scores, which approximate a *t*-statistic by scaling the minimum-norm estimate by a noise-sensitivity estimate at each source location. The noise covariance matrix was estimated from the pre-stimulus baseline interval (−100 to 0 ms). Individual source estimates were morphed to the fsaverage surface for group-level statistical inference. Statistical significance was assessed via a spatio-temporal cluster-based permutation test using 5000 permutations and a family-wise error (FWE)-corrected cluster-level significance threshold of α = 0.05 (two-tailed). Candidate clusters were formed using a threshold of *p* < 0.001 (corresponding to *t* > 3.57, df = 37). In this analysis, a cluster was defined as a set of spatially adjacent cortical vertices and temporally adjacent time points whose test statistics exceeded the cluster-forming threshold. Spatial adjacency between source vertices was defined based on the triangulated cortical surface mesh of the fsaverage template (oct6 spacing), ensuring that clusters reflect contiguous cortical patches rather than spatially disparate activations. The statistical significance of each cluster was evaluated against a permutation-derived null distribution of maximum cluster statistics, thereby controlling the family-wise error rate across the spatio-temporal search space. Regions of interest (ROIs) were defined based on the frequency of vertex occurrence within statistically significant clusters to characterize stable cortical activation patterns.

## 3. Results

### 3.1. ERP Results

Before presenting the neurophysiological results, we report the behavioral performance in the reversed speech paradigm. Participants demonstrated high attentional engagement, with a mean counting accuracy of 97.82% ± 4.37% (range: 77.5–100%), confirming that the active counting task was performed reliably across participants.

**Figure 3:**
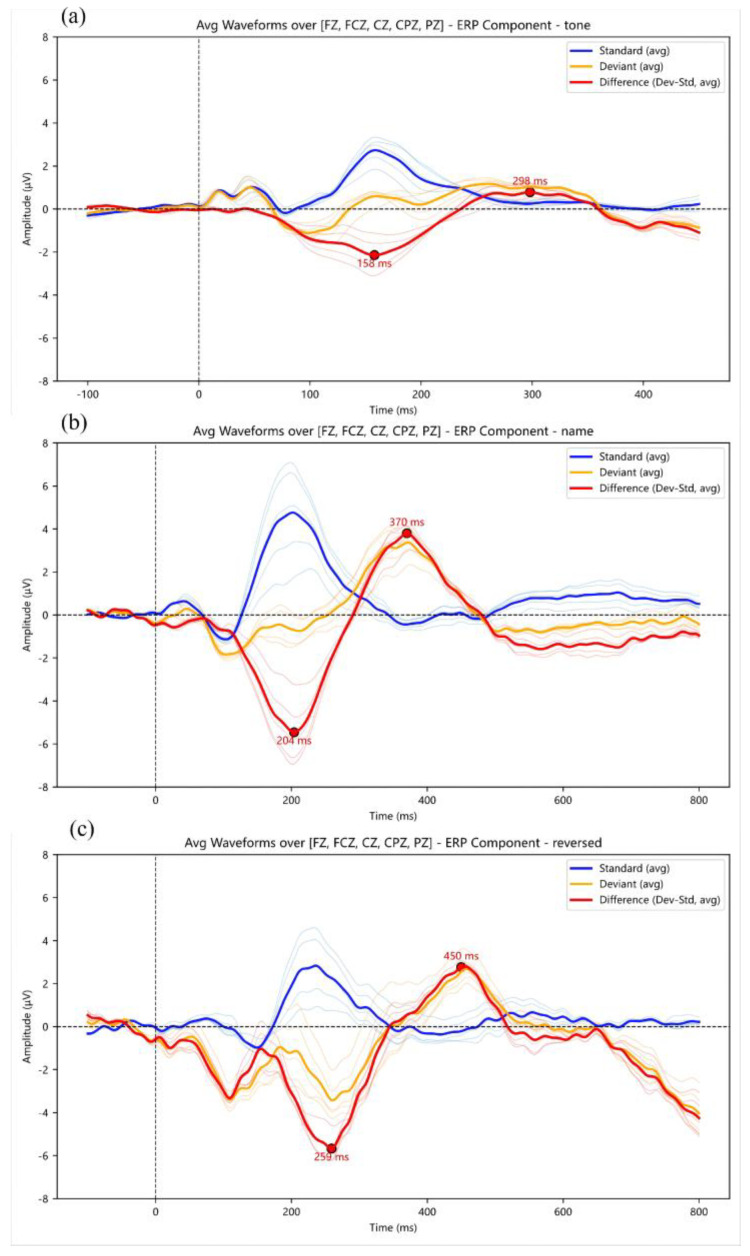
Grand-average ERP waveforms across the three auditory paradigms (Tone, Name, Reversed). Evoked responses were averaged across midline electrodes (Fz, FCz, Cz, CPz, Pz). For each paradigm, the standard (blue) and deviant (orange) waveforms are shown along with their difference wave (deviant minus standard; red). Shaded regions indicate ±1 standard error across participants. In the Tone paradigm (**a**), deviant tones elicited an early negative deflection (~150 ms) followed by a later positive component (~300 ms). In the Name paradigm (**b**), the participant’s own name induced a pronounced late positive component (~370 ms), reflecting enhanced self-relevant processing. In the Reversed paradigm (**c**), time-reversed speech elicited a negative deflection (~255 ms) and a later positive component (~450 ms), indicating auditory processing of acoustically complex but temporally disrupted speech signals with reduced semantic access. The waveforms illustrate paradigm-specific temporal dynamics in auditory deviance processing.

Grand-averaged ERP waveforms across the midline electrodes revealed that all three auditory oddball paradigms successfully elicited a biphasic neural response, consisting of an early negative deflection and a subsequent late positive component. As illustrated in Figure 3, the morphological characteristics of these ERP components were modulated by the type of auditory deviant stimulus. Detailed peak latencies and peak amplitudes for the MMN and P300 components are summarized in Table 1.

**Figure 4:**
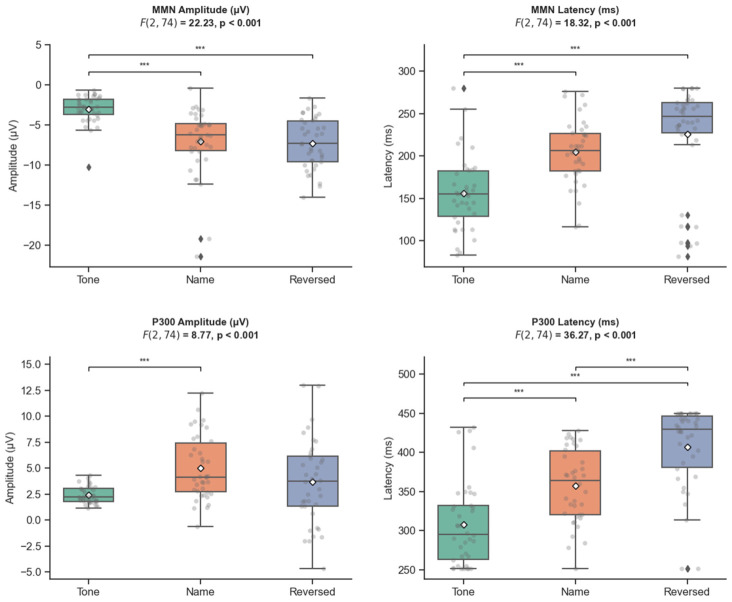
Statistical distribution of ERP components across three paradigms. (Tone, Name, Reversed). Each box plot displays the median (horizontal line), the mean (white diamond), and the interquartile range. Individual participant data points are overlaid as gray circles to show the distribution. Significant differences from post hoc Bonferroni-corrected comparisons are indicated by asterisks (*** *p* < 0.001).

The tone paradigm elicited the smallest and earliest responses, whereas the self-name paradigm induced the largest ERP signatures: the MMN reaching a peak amplitude of −6.39 µV at 205.0 ms and the P300 component reached a peak amplitude of 3.95 µV at 371.0 ms. Although the reversed speech paradigm yielded higher amplitudes than the tone paradigm, it exhibited the longest peak latencies across all temporal analysis windows, with the P300 component peaking at 450.0 ms. These findings indicate that while acoustic deviance is processed rapidly, self-referential stimuli elicit significantly larger and more discriminable neural responses. To further substantiate these observations, one-way repeated-measures ANOVA confirmed significant main effects of paradigm on all four ERP metrics (all *p* < 0.001), with large effect sizes throughout: MMN amplitude (F(2, 74) = 22.23, partial η^2^ = 0.375), MMN latency (F(2, 74) = 18.32, partial η^2^ = 0.331), P300 amplitude (F(2, 74) = 8.77, partial η^2^ = 0.192), and P300 latency (F(2, 74) = 36.27, partial η^2^ = 0.495). The detailed statistical distribution and results of the post hoc pairwise comparisons are illustrated in Figure 4.

Statistical comparisons of ERP features are summarized in Table 1 and Figure 4. For the MMN component, the Tone paradigm exhibited significantly shorter latencies and smaller amplitudes compared to both the Name and Reversed paradigms, highlighting the distinct neural encoding speed for simple versus complex auditory deviants. Regarding the P300 component, a significant main effect of paradigm was observed for latency across all three conditions. Specifically, the P300 latency followed a progressive increase from the Tone to Name, and finally to the Reversed paradigm. This hierarchical prolongation of latency likely reflects the increasing cognitive load and template-matching demands required to evaluate stimuli with higher acoustic and semantic complexity.

### 3.2. Source-Space Results

**Figure 5:**
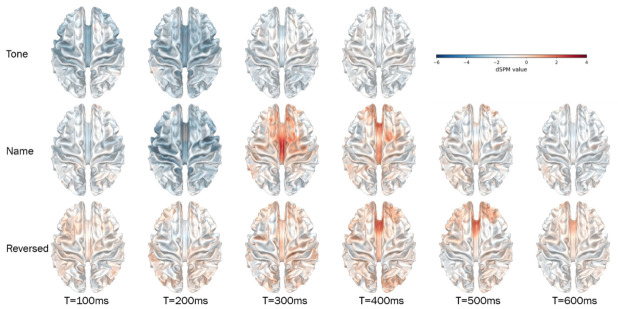
Spatio-temporal cortical activation maps reconstructed via dSPM. The cortical maps illustrate the dynamic neural activity for each paradigm (Tone, Name, Reversed) morphed to the fsaverage surface. The color scale denotes unitless dSPM values, which are noise-normalized statistical scores reflecting the strength of the neural response relative to the noise baseline. Warmer colors (positive values) indicate regions where the deviant condition elicited stronger activation than the standard condition, while cooler colors (negative values) indicate the reverse. These values serve as a standardized measure for comparing neural engagement across different temporal windows and paradigm variants.

Source-space estimation via dSPM was employed to map the scalp-recorded neural signals onto the cortical surface, revealing the spatio-temporal dynamics of auditory deviance processing. Figure 5 displays representative source-space activation maps at six post-stimulus time points (100, 200, 300, 400, 500, and 600 ms), whereas the full statistical cluster time ranges are reported in Table A1. For the Tone paradigm, a distinct neural engagement was visible at 200 ms, characterized by prominent negative (blue) dSPM values localized primarily to the bilateral superior temporal gyrus (STG), which became weaker at later displayed time points. In contrast, cortical recruitment for the Reversed Speech paradigm emerged primarily in the later processing stages, where positive (red) activation became evident at 300 ms and remained visible at the 400–600 ms time points, with the strongest signals concentrated in the temporal and cingulate regions.

The Name paradigm exhibited a clear two-phase transition in cortical activity across the displayed time points, beginning with negative (blue) activation in the bilateral temporal areas at 200 ms, followed by a rapid shift at 300 ms into stronger positive (red) activation. This positive activity was most prominent at the 300 and 400 ms time points. To substantiate these visual observations, a spatio-temporal cluster-based permutation test was performed on the reconstructed source data. This statistical analysis revealed varying levels of cortical engagement across the three variants: while the tone and reversed speech paradigms yielded 7 and 6 statistically significant clusters, respectively, the name paradigm exhibited the most extensive recruitment with 12 significant clusters. Detailed information regarding the statistical cluster time ranges, spatial topography, and statistical values of these clusters is summarized in Table A1.

### 3.3. Classification Results

**Figure 6:**
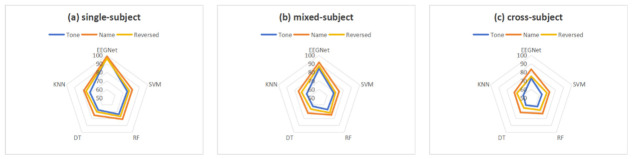
Classification performance for the three auditory paradigms (Tone, Name, Reversed) across five classifiers (SVM, RF, DT, KNN, EEGNet) under different evaluation schemes. (**a**) Single-subject: Models were trained and tested independently for each participant. (**b**) Mixed-subject: Data from all participants were pooled and randomly divided into training (70%) and testing (30%) sets. (**c**) Cross-subject: Models were trained on 70% of the participants and evaluated on the remaining 30%, repeated across subject splits. EEGNet consistently achieved the highest classification performance, and the Name paradigm yielded the strongest discriminability across evaluation schemes, followed by Reversed and Tone.

All classifiers achieved above-chance classification performance in distinguishing deviant from standard trials across the three paradigm variants. However, decoding accuracy exhibited consistent differences across both the three paradigm variants and different model types (Figure 6). Across all three evaluation schemes (single-subject, mixed-subject, and cross-subject), the self-name paradigm consistently exhibited the highest decoding performance, followed by the reversed speech and tone paradigms.

**Figure 7:**
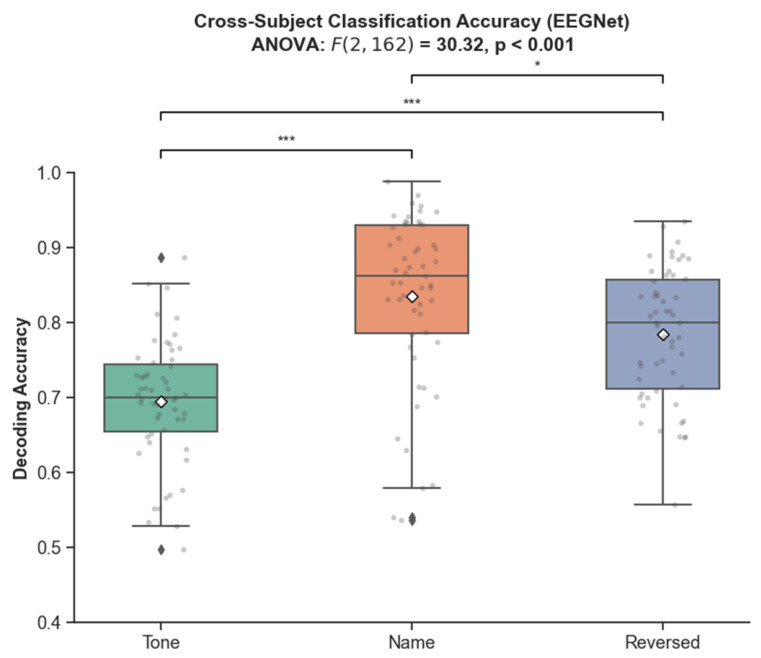
Cross-subject classification performance across three paradigms using EEGNet. The box plot illustrates the distribution of decoding accuracies for 11 test subjects across 5 independent repeat runs (n = 55 observations per paradigm). The horizontal line and white diamond within each box represent the median and mean values, respectively. Individual data points are overlaid to show the performance variance across subjects and runs. Significant differences between paradigms are indicated by asterisks (* *p* < 0.05, *** *p* < 0.001).

Among the five classifiers tested, the EEGNet model achieved the highest overall classification performance. Specifically, in the cross-subject condition of the self-name paradigm, which involved 55 independent test instances (5 repeats of 11-subject test sets), EEGNet reached a mean accuracy of 83.54% (SD = 11.37%), while the most competitive traditional model, SVM, yielded 72.03% (SD = 9.16%; independent samples *t*-test, t(108) = 5.84, *p* < 0.001). The calculated Cohen’s d was 1.11. In comparison, the RF, KNN, and DT models resulted in lower classification accuracies and showed higher variability across different test sets. To further evaluate the generalizability of neural representations elicited by different paradigms, cross-subject decoding results of the best-performing EEGNet model were subjected to statistical analysis. As shown in Figure 7, classification accuracies for all three paradigms were significantly above the theoretical chance level of 50% (binary classification of standard vs. deviant trials). Although a formal label-permutation test was not conducted, convergent evidence from five independent cross-subject runs with distinct subject partitions, a statistically significant one-way ANOVA (F(2, 162) = 30.32, *p* < 0.001), and consistent performance across both machine learning and deep learning classifiers collectively provide evidence against chance-level performance. Post hoc comparisons indicated that the self-name paradigm yielded higher decoding accuracy compared to both the tone and reversed speech paradigms, suggesting that self-referential stimuli elicit more individually consistent neural response patterns. The variance in cross-subject classification performance is illustrated in Figure 7, with SD values ranging from 9.16% (SVM) to 11.37% (EEGNet) in the self-name paradigm, reflecting moderate inter-individual variability typical of EEG-based decoding. Classification performance generally decreased as interindividual variability increased (i.e., single-subject > mixed-subject > cross-subject); however, the relative ranking of paradigm variants remained consistent across evaluation schemes, supporting the robustness of the observed paradigm differences. Since oversampling was applied consistently across all three paradigms, this invariant rank ordering (self-name > reversed speech > tone) remains interpretable as a reflection of differential neural discriminability across paradigm types, even if the absolute accuracy values should be interpreted with caution. The higher performance of the self-name paradigm in the cross-subject condition suggests greater standard-versus-deviant separability under the present recording and analysis conditions. These results indicate that self-referential auditory stimuli yielded higher decoding performance in the present offline analysis, suggesting their potential usefulness for future auditory ERP-based BCI paradigm optimization.

### 3.4. EEGNet Saliency Topography

**Figure 8:**
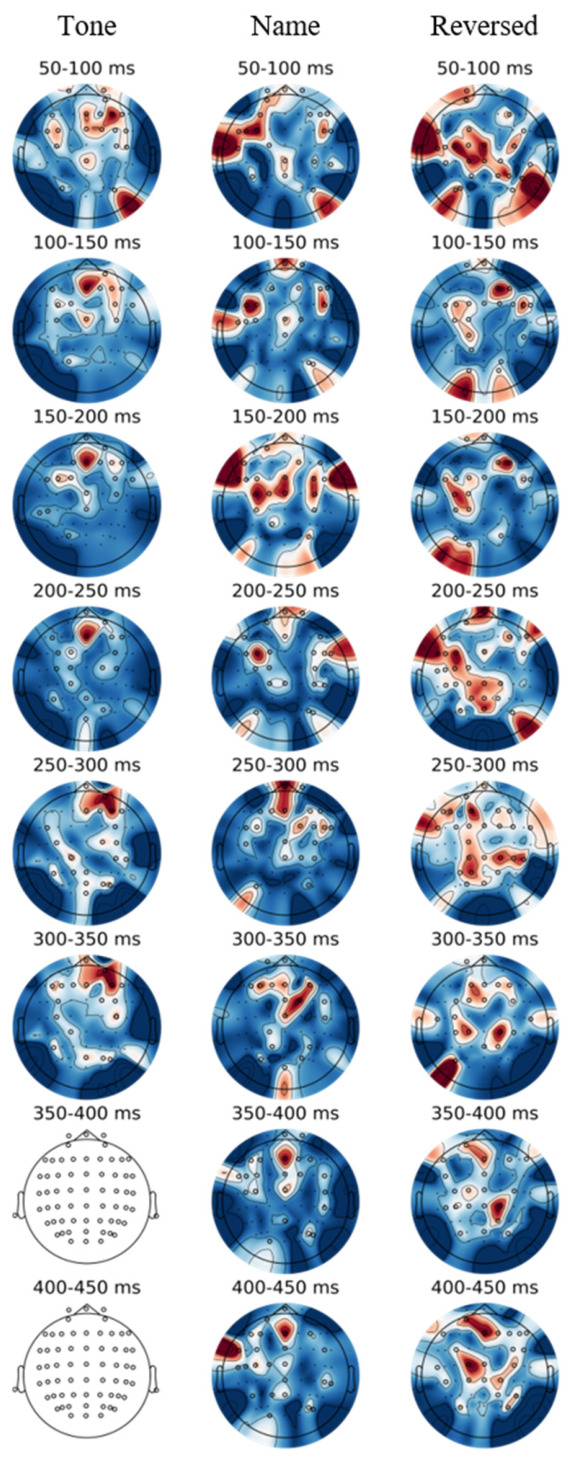
EEGNet saliency topography across paradigms. Saliency maps illustrate the group-averaged attribution scores derived from Integrated Gradients (IG) across three paradigms. Each row represents a 50 ms sliding window, and each column corresponds to a paradigm. Warmer colors indicate higher attribution values (standardized IG). Electrodes marked with white circles denote significant spatial contributions (*p* < 0.05), determined via a group-level one-sample permutation *t*-test (1000 iterations, one-tailed) followed by FDR correction. The null hypothesis assumes that an electrode’s contribution is no different from the global spatial mean. The results reveal progressively stronger and more spatially distributed discriminative patterns from tone to self-name paradigms, particularly within the 200–400 ms interval.

To further interpret the neural features driving EEGNet classification, spatiotemporal saliency maps derived from Integrated Gradients were analyzed as shown in Figure 8.

Across all three paradigms, discriminative neural patterns exhibited clear temporal evolution within the 50–450 ms post-stimulus interval. In the tone paradigm, salient activity was primarily confined to early time windows (50–150 ms), with spatial contributions localized to fronto-central regions. The overall saliency magnitude was relatively weak and spatially sparse, suggesting that EEGNet classification in the tone paradigm was mainly driven by early time-locked post-stimulus activity rather than broadly distributed discriminative patterns.

In contrast, the reversed speech paradigm demonstrated a broader temporal engagement, with salient patterns emerging from approximately 100 ms and extending into later stages (~350 ms). Spatially, these contributions involved both fronto-central and parietal regions, reflecting increased processing demands associated with structural reanalysis of speech.

The self-name paradigm exhibited the most prominent and sustained saliency patterns. Strong and widespread contributions were observed from 150 ms onward, peaking within the 200–400 ms interval. Notably, significant electrodes were distributed across fronto-central and parietal regions, consistent with the canonical P300 topography. Compared to the other paradigms, the self-name condition showed both higher saliency magnitude and greater spatial extent.

Importantly, the temporal windows showing maximal saliency closely aligned with ERP components identified in Section 3.1, particularly the MMN and P300 intervals. Furthermore, the spatial distribution of salient electrodes corresponded with cortical regions identified in source-space analysis (Section 3.2), suggesting that EEGNet classification is primarily driven by neurophysiologically meaningful patterns rather than spurious features.

Collectively, these results suggest that increasing stimulus relevance is associated with larger response amplitudes and more spatially distributed discriminative neural representations captured by the EEGNet model.

## 4. Discussion

The present study provides a multi-level comparison of neural responses across three auditory oddball paradigm variants, revealing a systematic hierarchy in neural engagement as a function of deviant stimulus relevance. All three paradigm variants elicited clear deviant-related neural responses to deviant stimuli, confirming the effectiveness of the auditory oddball paradigm for investigating auditory change detection [50,51]. However, notable differences in neural response were observed across the three paradigms. The self-name condition produced larger P300 amplitudes, more extensive cortical activation patterns, and higher classification accuracy compared with the tone and reversed speech conditions. In particular, the P300 amplitude in the self-name condition was approximately 3.9 μV, whereas the tone condition elicited a markedly smaller response of approximately 1.8 μV. These findings demonstrate that self-referential auditory stimuli elicit stronger and more discriminable neural responses than other types of auditory deviant stimuli.

Before interpreting these differences, it is important to note that the three paradigms differed not only in deviant type but also in standard stimuli, stimulus probability ratios, inter-stimulus intervals, trial counts, stimulus durations, and task demands. These design differences may have influenced the observed ERP and classification results. For example, differences in standard stimuli may alter the sensory memory trace against which deviants are compared, thereby affecting MMN-related responses [1]. Differences in trial counts may influence ERP signal-to-noise ratio, statistical power, and the stability of classification estimates, even though class balancing was applied during model training [52]. In addition, active counting in the reversed speech paradigm may increase attentional allocation and modulate P300 amplitude relative to passive listening [2]. These differences reflect the naturalistic design constraints of each paradigm variant and mean that the observed neural differences cannot be attributed solely to stimulus type. Accordingly, the following discussion frames the results as a comparative characterization of three commonly used paradigm variants rather than as causal evidence for the unique contribution of deviant identity alone.

These observed differences across the three paradigm variants may be attributed to the distinct intrinsic characteristics of the deviant stimuli. Although the enhanced neural response to one’s own name has been documented in earlier ERP studies, the present work extends these findings in several key dimensions. In the tone oddball paradigm, deviant stimuli are typically defined by simple acoustic changes (e.g., frequency differences). This paradigm primarily engages the basic neural mechanisms of auditory change detection and has been widely used to investigate early stages of auditory sensory processing [53]. While earlier investigations primarily established the “own-name effect” by comparing self-referential stimuli with such simple sinusoidal tones, our study situates the self-name paradigm within a broader cognitive hierarchy by comparing it also to reversed speech—a complex auditory signal requiring significant structural and semantic reanalysis. The fact that the self-name elicited larger and more discriminable neural patterns than even the complex reversed speech stimuli supports the interpretation that self-referential information has high cognitive priority over general semantic complexity. Processing one’s own name is known to capture automatic attentional resources and elicit enhanced neural responses, even under conditions of limited attentional resources. Consistent with previous research findings [21,22,23], the present results showed that self-name stimuli yielded the largest P300 responses among the three paradigm variants. Moreover, whereas previous research predominantly relied on manual ERP feature extraction, our integration of 3D cortical source localization and EEGNet-based explainability analysis provides a more comprehensive, data-driven validation of these classic neurophysiological markers, linking temporal ERP components to their underlying spatial neural generators.

Beyond the observed ERP differences, the classification analysis further confirmed that neural responses to self-name stimuli were more readily distinguishable from those to standard stimuli [54]. Classification accuracy in the self-name condition reached approximately 80%, which was markedly higher than that in the tone and reversed speech paradigms. This finding indicates that self-referential auditory stimuli elicit neural responses with significantly higher discriminability. However, higher decoding accuracy should not be interpreted as direct evidence for richer or more complex neural representations. Classification performance can benefit from larger ERP amplitudes, higher signal-to-noise ratio, and more consistent time-locked responses, particularly for components such as the P300. Therefore, the superior decoding performance of the self-name paradigm is more conservatively interpreted as greater standard-versus-deviant separability under the present recording and analysis conditions.

The potential translational significance of these findings lies primarily in paradigm optimization rather than immediate clinical deployment. In clinical assessment research, self-name stimuli may provide a personally salient auditory probe for evaluating residual auditory attention and self-referential processing in individuals with limited behavioral responsiveness, such as patients with disorders of consciousness or severe communication impairment. Because the response can be elicited under passive listening, this paradigm may be particularly useful when overt behavioral responses are unreliable or unavailable. In auditory ERP-based BCI research, self-name stimuli may serve as personalized target stimuli to enhance target/non-target separability, especially in situations where visual stimulation is impractical. However, these applications remain prospective: future studies should validate the paradigm in target clinical populations and online BCI settings before any diagnostic or practical BCI use can be claimed.

To further bridge the gap between neural representation and model performance, the EEGNet-based saliency analysis provides critical insights into the features underlying successful classification. The saliency maps revealed that EEGNet predominantly relied on temporally and spatially structured neural activity rather than diffuse or noise-driven patterns.

Specifically, the alignment between saliency peaks and canonical ERP components, such as the MMN and P300, suggests that the model implicitly captures well-established neurophysiological signatures of auditory deviance processing. The spatial distribution of salient electrodes identified by the IG algorithm aligns with established scalp topographies associated with auditory attention and self-referential processing.

The fronto-central prominence of saliency peaks is consistent with the topographic distribution of the Mismatch Negativity (MMN) and P3a components, which are indices of automatic deviance detection and involuntary attention switching. In the self-name paradigm, the significant involvement of parietal regions mirrors the scalp topography of the P3b (or late positive component), a marker typically associated with the allocation of attentional resources to task-relevant or personally significant stimuli [2]. Furthermore, this parietal and midline-centric distribution overlaps with the ‘cortical midline structures’ (CMS) identified as core regions for processing self-related information [55].

However, certain discrepancies between the IG-based saliency maps and traditional grand-average ERP topographies should be noted. While traditional ERP maps often exhibit broad, diffuse activation due to linear averaging across subjects, the IG maps in this study manifested a more focal and localized distribution. This discrepancy likely suggests that the deep learning model prioritizes a subset of highly discriminative spatial features that maximize classification accuracy, rather than reflecting the entire physiological field typically captured by standard topographic mapping.

Importantly, the progressive increase in saliency strength and spatial extent from tone to reversed speech to self-name paradigms mirrors the hierarchy observed in ERP amplitude, source-level activation, and classification performance. This convergence across analytical levels supports the interpretation that enhanced behavioral relevance is associated with more spatially coherent, and temporally stable neural representations, which are more readily captured by deep learning models.

From a methodological perspective, these findings highlight that EEGNet does not function as a “black box” but rather extracts physiologically meaningful features that correspond to known cognitive processes. The integration of explainability analysis thus strengthens the interpretability of decoding results by relating model-relevant features to known ERP time windows and scalp topographies.

A critical methodological consideration in the present study is the variation in deviant-to-standard ratios and inter-stimulus intervals (ISI) across the three paradigms. Specifically, the deviant ratios were 1:9 for the tone, 1:3 for the self-name, and 1:2 for the reversed speech. According to the principles of stimulus-specific adaptation (SSA) and predictive coding, lower-probability stimuli (rarer deviants) typically elicit stronger neural responses due to reduced habituation and increased “surprise.” However, the observed neural response patterns in this study did not strictly follow the inverse relationship predicted by SSA. Despite being more frequent (1:3) than the pure tone deviants (1:9), the self-name elicited a markedly larger P300 amplitude and more extensive cortical recruitment. This discrepancy suggests that the biological and social salience of the self-name provides a substantial contribution to the neural response that extends beyond simple probability-based deviance detection. While SSA likely influenced the overall magnitude of the ERP components, the unique spatiotemporal signatures suggest that the intrinsic semantic salience of the stimulus provides a distinct and meaningful contribution to the neural response that is not easily explained by stimulus probability alone.

Furthermore, the task-related requirements differed across conditions, with participants performing an active counting task during the reversed speech paradigm while remaining in a passive listening state for the other two variants. This adjustment was necessitated by the higher cognitive load inherent in processing non-intelligible speech structures. Theoretically, such active engagement is expected to significantly enhance the P3 component by facilitating additional attentional resource allocation. However, an important observation in our data is that despite this deliberate attentional ‘boost’ provided to the reversed speech condition, it still failed to elicit neural responses or classification performance comparable to the self-name paradigm conducted under purely passive conditions. This comparison suggests that the intrinsic biological salience of self-referential stimuli is a more potent driver of neural engagement than explicit task-directed attention, further reinforcing our conclusion regarding the cognitive priority of the self-name. Nevertheless, because stimulus probability, trial number, acoustic structure, and task instruction were not fully matched across paradigms, the present data cannot isolate stimulus salience from task demand or probability-related effects. Therefore, the observed differences should be interpreted as reflecting the combined influence of self-relevance, acoustic/semantic complexity, probability structure, and task engagement rather than the effect of stimulus salience alone.

The high counting accuracy observed in the reversed speech paradigm (97.82% ± 4.37%) further confirms that this attentional engagement was genuine and consistent across participants, ruling out the possibility that the relatively weaker neural responses in this condition were attributable to insufficient task compliance. This distinction aligns with Polich’s theoretical framework differentiating P3a, which reflects automatic stimulus-driven attention capture, from P3b, which reflects voluntary task-directed resource allocation. Within this framework, the MMN and early P3a-like responses observed across all three paradigms likely reflect automatic deviance detection processes, whereas the larger P3b observed in the self-name paradigm suggests that self-referential stimuli engage a more profound and sustained mobilization of attentional resources. Crucially, this enhanced P3b emerged under passive listening conditions, suggesting that the attentional capture by one’s own name operates largely automatically, consistent with previous evidence that self-referential stimuli possess privileged access to attentional resources [8].

Nevertheless, several limitations should be acknowledged when interpreting these findings. First, while the results suggest a primary role for stimulus salience, the lack of strictly equalized presentation parameters—including differences in standard stimuli, deviant ratios, trial counts, inter-stimulus intervals, stimulus durations, and the shift between active and passive tasks—remains a limitation. Specifically, the inclusion of a counting task in the reversed speech paradigm introduces a potential confounding effect of task-directed attention. Although our analysis suggests that the intrinsic salience of the self-name outweighs these task-related enhancements, the present study does not constitute a strictly isolated comparison of stimulus complexity or task demand across all conditions. Instead, the results should be interpreted as a comparative analysis of commonly used auditory oddball paradigm variants in their optimized forms.

Second, the three paradigms differed in multiple acoustic properties beyond their behavioral relevance. Consequently, as the physical parameters were not perfectly matched across the tone, reversed speech, and self-name deviants, this study does not constitute a strictly controlled comparison of the stimuli’s intrinsic properties. The observed neural differences thus reflect the composite effect of these diverse stimulus characteristics and their respective cognitive processing requirements.

Third, the present study only included healthy young adult participants. Given that auditory change detection and self-referential processing may be altered in neurological or psychiatric conditions, the generalizability of these findings to clinical or older adult populations remains to be explored. Future research utilizing acoustically balanced controls and broader demographic samples is warranted to more precisely disentangle the relative contributions of stimulus probability, acoustic complexity, and intrinsic salience.

Finally, a formal label-permutation test for classifier performance was not conducted in the present study. Although convergent evidence from repeated independent cross-subject runs and statistically significant group-level comparisons provides support against chance-level performance, future work should include permutation-based null distributions to provide more rigorous statistical validation of classification results.

## 5. Conclusions

The present study compared neural responses across three variants of the auditory oddball paradigm, which incorporated tone, reversed speech, and self-name deviant stimuli. All three paradigm variants successfully elicited clear deviant stimulus-related neural responses, demonstrating the effectiveness of the auditory oddball paradigm design for investigating auditory change detection in the human brain.

Among the three paradigms, self-name stimuli yielded significantly larger P300 amplitudes, more extensive cortical activation patterns, and higher classification accuracy than tone and reversed speech deviant stimuli. These findings suggest that self-referential auditory stimuli may elicit significantly stronger and more discriminable neural responses in the auditory oddball paradigm. These results provide a valuable comparative perspective on neural responses to different types of auditory deviant stimuli and contribute to the selection and design of effective auditory oddball paradigms for cognitive neuroscience research and translational applications. Importantly, the convergence of ERP, source-space, classification, and saliency analyses provides a multi-level validation framework, demonstrating that deep learning models can rely on neurophysiologically meaningful EEG patterns associated with auditory salience.

## Figures and Tables

**Table 1:** Grand-average ERP Peak Latencies (ms) and Amplitudes (μV) for MMN and P300 Components.

Paradigm	MMN_Latency	MMN_Amplitude	P300_Latency	P300_Amplitude
Tone	158.0	−2.30	310.0	1.77
Name	205.0	−6.39	371.0	3.95
Reversed	259.0	−5.67	450.0	2.55

Note: MMN metrics were derived from difference waveforms (deviant minus standard), while P300 metrics were quantified directly from deviant stimulus trials. The unit for the latency indicator is milliseconds, and the unit for the amplitude indicator is microvolts.

## Data Availability

The data supporting this study’s findings are available on request with the consent of the corresponding authors F.D. However, the data are not publicly available because they contain information that could compromise the privacy of research participants.

## Appendix A

Appendix A presents the source-space cluster results obtained using the spatio-temporal permutation test.

**Table A1:** Summary of significant spatio-temporal clusters for Tone, Reversed, and Name paradigms.

Paradigm	Cluster ID	Time Range (ms)	Vertices	*p*-Value	Primary Regions
Tone	0	1.0–395.0	3889	0.0003	superiorfrontal-lh: 278 (7.1%); precentral-lh: 256 (6.6%); superiorparietal-lh: 239 (6.1%); postcentral-lh: 202 (5.2%); rostralmiddlefrontal-lh: 201 (5.2%)
	1	5.0–450.0	3748	0.0003	inferiorparietal-rh: 241 (6.4%); superiorfrontal-rh: 238 (6.4%); superiorparietal-rh: 229 (6.1%); precentral-rh: 220 (5.9%); rostralmiddlefrontal-rh: 197 (5.3%)
	2	37.0–107.0	97	0.0180	superiorparietal-lh: 50 (51.5%); lateraloccipital-lh: 26 (26.8%); inferiorparietal-lh: 21 (21.6%)
	3	134.0–208.0	52	0.0233	superiorparietal-rh: 40 (76.9%); inferiorparietal-rh: 12 (23.1%)
	4	351.0–398.0	70	0.0410	inferiorparietal-rh: 35 (50.0%); superiorparietal-rh: 35 (50.0%)
	5	360.0–450.0	1920	0.0007	precentral-lh: 159 (8.3%); superiortemporal-lh: 140 (7.3%); insula-lh: 118 (6.1%); postcentral-lh: 107 (5.6%); precuneus-lh: 100 (5.2%)
	6	401.0–430.0	74	0.0350	superiorparietal-rh: 74 (100.0%)
Name	0	322.0–397.0	61	0.0127	lateralorbitofrontal-lh: 5 (8.2%); medialorbitofrontal-lh: 1 (1.6%)
	1	34.0–101.0	117	0.0167	insula-lh: 64 (54.7%) parstriangularis-lh: 20 (17.1%); parsopercularis-lh: 11 (9.4%); superiortemporal-lh: 9 (7.7%); supramarginal-lh: 8 (6.8%)
	2	49.0–118.0	69	0.0260	supramarginal-rh: 43 (62.3%); postcentral-rh: 16 (23.2%); insula-rh: 9 (13.0%); precentral-rh: 1 (1.4%)
	3	134.0–208.0	151	0.0083	posteriorcingulate-lh: 48 (31.8%); caudalanteriorcingulate-lh: 7 (4.6%); isthmuscingulate-lh: 2 (1.3%)
	4	60.0–114.0	120	0.0153	posteriorcingulate-rh: 22 (18.3%); caudalanteriorcingulate-rh: 12 (10.0%); rostralanteriorcingulate-rh: 1 (0.8%)
	5	111.0–270.0	1913	0.0003	superiortemporal-rh: 128 (6.7%); precentral-rh: 117 (6.1%); precuneus-rh: 111 (5.8%); superiorparietal-rh: 106 (5.5%); supramarginal-rh: 101 (5.3%)
	6	121.0–265.0	1974	0.0003	precentral-lh: 211 (10.7%); postcentral-lh: 171 (8.7%); superiorfrontal-lh: 117 (5.9%); superiortemporal-lh: 111 (5.6%); precuneus-lh: 109 (5.5%)
	7	151.0–249.0	30	0.0340	inferiorparietal-rh: 30 (100.0%)
	8	153.0–235.0	22	0.0367	precentral-rh: 22 (100.0%)
	9	630.0–764.0	44	0.0150	precentral-lh: 21 (47.7%); parsopercularis-lh: 14 (31.8%); caudalmiddlefrontal-lh: 8 (18.2%); rostralmiddlefrontal-lh: 1 (2.3%)
	10	630.0–706.0	70	0.0443	insula-rh: 46 (65.7%); supramarginal-rh: 13 (18.6%); postcentral-rh: 7 (10.0%); superiortemporal-rh: 3(4.3%); precentral-rh: 1 (1.4%)
	11	675.0–707.0	106	0.0170	medialorbitofrontal-rh: 5 (4.7%); rostralanteriorcingulate-rh: 1 (0.9%)
Reversed	0	422.0–508.0	46	0.0137	caudalanteriorcingulate-lh: 29 (63.0%); rostralanteriorcingulate-lh: 6 (13.0%); posteriorcingulate-lh: 3 (6.5%); superiorfrontal-lh: 2 (4.3%)
	1	724.0–800.0	58	0.0100	none
	2	730.0–800.0	41	0.0180	posteriorcingulate-rh: 27 (65.9%); precuneus-rh: 14 (34.1%)
	3	731.0–800.0	53	0.0037	posteriorcingulate-lh: 22 (41.5%); isthmuscingulate-lh: 12 (22.6%); caudalanteriorcingulate-lh: 1 (1.9%)
	4	733.0–808.0	120	0.0023	posteriorcingulate-rh: 24 (20.0%); isthmuscingulate-rh: 16 (13.3%); caudalanteriorcingulate-rh: 2 (1.7%)
	5	205.0–275.0	35	0.0283	precentral-rh: 31 (88.6%); caudalmiddlefrontal-rh: 2 (5.7%); parsopercularis-rh: 2 (5.7%)

Note: Statistical significance was determined using a non-parametric spatio-temporal cluster-based permutation test (5000 iterations, two-tailed) based on a sample size of n = 38. The cluster-forming threshold was statistically defined at a significance level of *p* < 0.001 to ensure high spatial and temporal specificity, while the final cluster-level significance was assessed using a family-wise error (FWE) corrected threshold of alpha = 0.05.
